# Tracing Potential Covalent Inhibitors of an E3 Ubiquitin Ligase through Target-Focused Modelling

**DOI:** 10.3390/molecules24173125

**Published:** 2019-08-28

**Authors:** Imane Bjij, Pritika Ramharack, Shama Khan, Driss Cherqaoui, Mahmoud E. S. Soliman

**Affiliations:** 1 Molecular Bio-Computation & Drug Design Lab, School of Health Sciences, University of KwaZulu-Natal, Westville, Durban 4000, South Africa; 2 Département de Chimie, Faculté des Sciences Semlalia, Université Cadi Ayyad, Av. My Abdellah, BP2390 Marrakech, Morocco; 3 Department of Clinical Microbiology and Infectious Diseases, School of Pathology, University of the Witwatersrand, Johannesburg 2193, South Africa

**Keywords:** covalent inhibition, NEDD4-1 E3 ligase, molecular modeling, pharmacophore modeling, molecular dynamic simulations

## Abstract

The Nedd4-1 E3 Ubiquitin ligase has been implicated in multiple disease conditions due its overexpression. Although the enzyme may be targeted both covalently and non-covalently, minimal studies provide effective inhibitors against it. Recently, research has focused on covalent inhibitors based on their characteristic, highly-selective warheads and ability to prevent drug resistance. This prompted us to screen for new covalent inhibitors of Nedd4-1 using a combination of computational approaches. However, this task proved challenging due to the limited number of electrophilic moieties available in virtual libraries. Therefore, we opted to divide an existing covalent Nedd4-1 inhibitor into two parts: a non-covalent binding group and a pre-selected α, β-unsaturated ester that forms the covalent linkage with the protein. A non-covalent pharmacophore model was built based on molecular interactions at the binding site. The pharmacophore was then subjected to virtual screening to identify structurally similar hit compounds. Multiple filtrations were implemented prior to selecting four hits, which were validated with a covalent conjugation and later assessed by molecular dynamic simulations. The results showed that, of the four hit molecules, Zinc00937975 exhibited advantageous molecular groups, allowing for favourable interactions with one of the characteristic cysteine residues. Predictive pharmacokinetic analysis further justified the compound as a potential lead molecule, prompting its recommendation for confirmatory biological evaluation. Our inhouse, refined, pharmacophore model approach serves as a robust method that will encourage screening for novel covalent inhibitors in drug discovery.

## 1. Introduction

The main class of E3 ubiquitin ligases are enzymes that constitute a HECT (homologous to E6-AP carboxyl terminus) domain [1,2]. These enzymes play an important role in the ubiquitination process, by transferring protein substrates to ubiquitin [3,4]. The Neural precursor cell Expressed Developmentally Down-regulated gene 4-1 (Nedd4-1) ubiquitin ligase is one of the Nedd4 enzymes that uses the HECT domain in the ubiquitination process [5]. In addition to the HECT region incorporated in the C-terminal domain, Nedd4-1 contains two other domains: the N-terminal domain and the multiple WW domain (double tryptophan residues) [6]. When overexpressed, Nedd4-1 alters normal metabolic processes, thereby implicating the enzyme in the pathogenesis of many human cancers [7,8].

The Nedd4-1 enzyme comprises a HECT domain that contains two shallow binding sites enclosing two cysteine residues. While the first is a catalytic site cysteine (Cys^867^), the second forms part of the allosteric site (Cys^627^) [9]. The presence of these nucleophilic residues allow covalent inhibition of the enzyme when bound to an electrophilic moiety of the inhibitor. The catalytic inhibition of the enzyme blocks the substrate from binding by occupying its active site [10]. However, allosteric inhibition can halt substrate binding by altering one or more of the kinetic parameters that define the properties of the catalytic site and the implicated biological activity of the protein [11,12]. Experimental studies as well as computational results from previous reports exhibit the selectivity of a covalent inhibitor toward the allosteric site over the binding to the catalytic site of Nedd4-1 [9,13]. This prompted us to focus our study on the allosteric site of this enzyme and generate a pharmacophore model based on these results.

The pace and efficiency of identifying active chemical entities most likely to interact with a target protein encapsulates the process of drug discovery and development. Hence, the emanation and prominence of virtual screening as an in silico approach is needed for the improvement of drug discovery. Virtual screening (VS) is a hit identification technique that automatically screens and evaluates an enormous library of chemical compounds to appropriately identify similar compounds based on structural complementarities. Various protocols and tools are available to screen databases for these drug compounds. Our approach includes different computational methods that will allow us to filter virtual compound libraries to discover novel covalent inhibitors of Nedd4-1. Our combinatorial strategy includes pharmacophore model generation, molecular docking, molecular dynamic simulations, and ADME (Absorption, Distribution, Metabolism, and Excretion) profile analysis.

**Figure 1:**
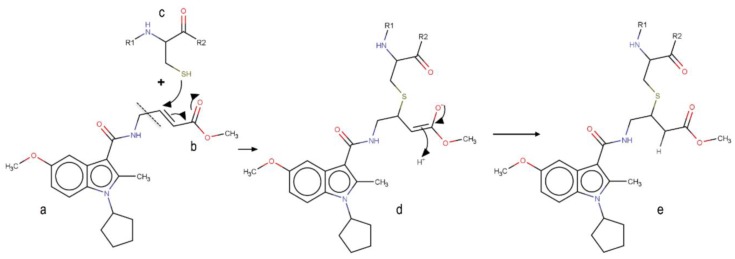
Mechanism of the covalent binding to the allosteric cysteine of Nedd4-1. **a** Non-covalent binding part of the inhibitor. **b** Covalent warhead of the inhibitor (α, β-unsaturated ester). **c** Allosteric cysteine (Cys^627^) of the Nedd4-1. **d** Intermediate product of the reaction. **e** Product of the reaction.

Although covalent compounds have proved to be promising in the inhibition of Nedd4-1, literature elucidating the virtual, screened covalent inhibitors is limited. This may be a result of the structural peculiarities of these compounds, including particular fragments that are responsible for the covalent linkage with a corresponding amino-acid residue of a protein. Identification of covalent hits or lead compounds in drug discovery requires proper optimization of both the covalent and non-covalent group of the ligand. In this study, we opted to split the covalent inhibitor into two parts: the group making up the covalent bond and the fragment responsible for the other non-covalent interactions stabilizing the ligand inside the complex, as seen in Figure 1. The α, β-unsaturated ester was chosen as the covalent part of the model; the non-covalent part was attained by virtual screening.

This study aimed to achieve covalent hits of unique chemical compounds that generate a novel pharmacological profile in the inhibition of Nedd4-1. Due to the challenges related to this task and the scarcity of the electrophilic moieties in the chemical libraries, we elected to use the non-covalent part of an existing covalent inhibitor of Nedd4-1 to generate a pharmacophore model and screen for novel inhibitor scaffolds of this enzyme.

## 2. Material and Methods

### 2.1. Structure Preparation and Pharmacophore Model Generation

Structure of Nedd4-1 was obtained from the Protein Data Bank (PBD), in a PDB format, under the code 5C91. The co-crystallized ligand, water molecules, and other co-factors were removed from the PDB structure using UCSF Chimera software. The structure of the ligand (methyl(2*E*)-4-[(1-cyclopentyl-5-methoxy-2-methylindol-3-yl)formamido]but-2-enoate) [9] was drawn in mol2 format using MarvinSketch 6.2.1, 2014, ChemAxon (www.chemaxon.com); hybridization state and proper angles were assessed using Molegro Molecular Viewer (MMV) [14].

During the study, the scarcity of compounds with electrophilic moiety in the available virtual libraries was significantly challenging. To overcome this obstacle, we manually removed the covalent binding region (the α, β-unsaturated ester) from the inhibitor for later attachment. In other words, the initial inhibitor was split into two sections: a warhead region responsible for the covalent attachment with the enzyme and a non-covalent region that is in charge of other interactions important for the occurrence of biological inhibition. This non-covalent section of the inhibitor was used in the screening for new hits of the Nedd4-1.

**Figure 2:**
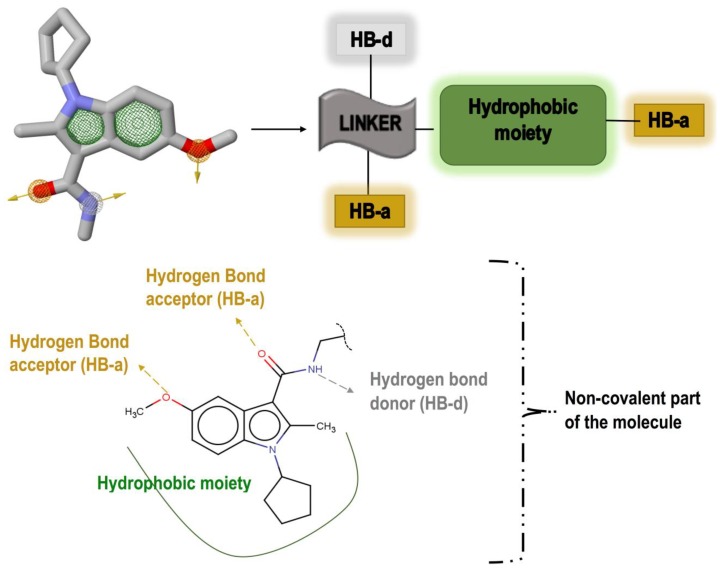
Important pharmacophoric features of the inhibitor’s non-covalent part and generation of a pharmacophore model based on these features.

To have an overall conformational picture rather than a simple ligand-based approach, our objective was to include all the interactions existing between the allosteric site and the ligand in our pharmacophore model. Therefore, the pharmacophore model was generated based on the non-covalent interactions demonstrated in our recent study, which analysed the allosteric site of Nedd4-1 when bound to the aforementioned covalent ligand [13]. These interactions occurred between the ligand and the allosteric site occupying residues PRO 603, TYR 604, TYR 605, GLY 606, ASN 621, ASN 623, LEY 626, CYS 627, and PHE 707. Important interactions were observed between TYR 605 and the hydrophobic moiety of the ligand as well as between the ligand and PRO 603 and LEU 626. To achieve the attributes of the non-covalent binding part, the ZincPharmer online tool was used to detect key interactions and then to automatically create an advanced pharmacophore model for virtual screening. The major interactions of this model, as shown in Figure 2, were common non-covalent hydrogen-bond donors and acceptors as well as the hydrophobic moiety of the ligand. The created model was then uploaded to ZINC database, thereby identifying 3304 hit molecules that were later saved in Structure Data File (SDF) format. The file was downloaded and converted to MOL2 format using the Open Babel tool [15]. The geometry and molecular energy of the hits were minimized using the semi-empirical method, AM1, implemented in PyRx [16].

### 2.2. Molecular Docking and Virtual Screening

Prior to the virtual screening, molecular docking of the initial inhibitor (methyl(2*E*)-4-[(1-cyclopentyl-5-methoxy-2-methylindol-3-yl)formamido]but-2-enoate) at the allosteric site of the Nedd4-1 was conducted. This procedure allowed for the verification of the docking parameters used during screening. The Autodock Vina tool [17] was used for all docking procedures in this study. Although the process was run with default parameters of the software, the exhaustiveness was fixed to 8 and the grid box was defined, using AutoDock tools GUI [18], around the above-mentioned key amino acid residues of the allosteric site. Docking calculations were conducted using the Lamarckian genetic algorithm within Autodock Vina. The grid box size and center parameters for the allosteric site were x (−15, 35) y (20, 5) and z (20, −5), respectively.

Following pharmacophore-based screening, docking of the generated database (3304 compounds) was executed using the AutoDock Vina plug-in in Pyrx [16]. Subsequently, the top 114 compounds were selected for eventual covalent warhead attachment, based on the favorable binding affinities. Ligand and enzyme modification as well as visualizations were performed in UCSF Chimera software package [19] and Maestro software [20].

### 2.3. Attachment of Covalent Binding Part 

The generation of inhibitors with electrophilic groups from virtual libraries is extremely scarce. For instance, when the α, β-unsaturated ester group was defined as a screening feature, no hits were identified, and thus no novel backbones could be explored. This augmented the challenge of screening for a covalent binding group using databases. One approach for evading this drawback is to pre-select the covalent feature by manually defining the electrophilic fragment and search for novel non-covalent backbones. In this report, α, β-unsaturated ester was defined as a covalent-binding feature, which would eventually be attached to the screened hits. We believe that our approach can be beneficial in the search of α, β-unsaturated esters with novel backbones.

According to the initial pharmacophore model α, β-unsaturated ester can be annexed close to the hydrogen bond acceptor (HB-a) and the hydrogen bond donor (HB-d). However, a methyl group is still required to eliminate any possible steric clashes between atoms as well as to secure the proper direction of covalent warhead attachment.

**Figure 3:**
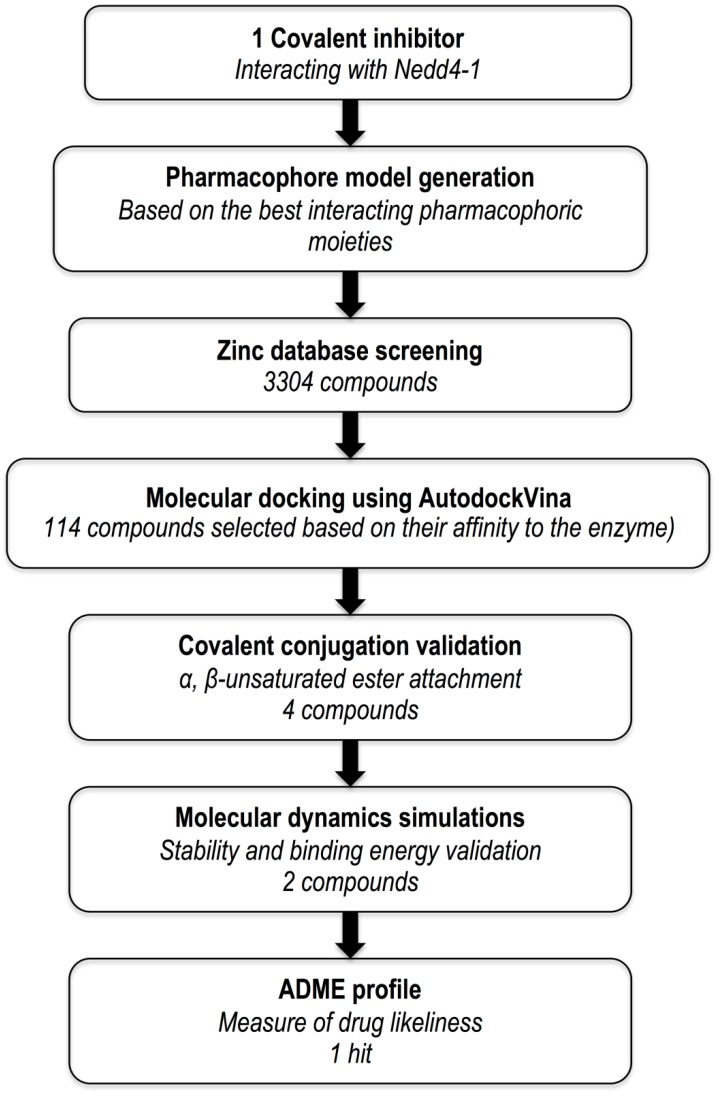
Outline of computational protocol and implemented filters.

The following filters were implemented in the selection of compounds for possible covalent appending: (a) minimal steric hindrance near the HB-a and the HB-a of the structures (preferably the presence of a methyl group) to allow the covalent fragment to hit in the right directions; (b) The excluded volumes were used to eliminate compounds likely to have steric conflicts with the protein; and (c) Compounds with the highest binding affinity were retained in a group of candidates with the same scaffold. One of the major objectives was to save the original chirality of the compounds and cause no conflicts between the atoms during attachment. The molecules that met the required conditions were then selected for electrophilic amendment. The prepared system’s protonation states were optimized, hydrogens were adjusted, and capping residues (acetyl and methylamide) were added to the covalent system with the Protein Preparation Wizard in Maestro Schrodinger [20]. The protocol of the overall procedure is represented in Figure 3.

### 2.4. Molecular Dynamic Simulations of Covalent Complex

Prior to the molecular dynamic (MD) simulations, the covalent ligand and the allosteric cysteine residue were parameterized to define atom types and partial charges using Antechamber module [21] in AmberTools14 suite of programs [22]. LEaP module was used to build a library defining the covalent residue topology. Dabble [23] was used to create, solvate, and neutralize (three Na+ counterions) the final input co-ordinate files to run MD. For the covalent system, we followed the computational methodology from our in-house protocol reported previously [13]. The numbering of the residue sequences in the studied complexes was automatically modified conforming to Amber format, leading to a new numbering arrangement. Residue sequence numbering of the simulated complexes starts at 1 and ends at 377 (counting the two capping residues); however, the original pdb structure starts at 519 and ends at 893. In Table 1, we present the original residue numbering of the allosteric site obtained from the crystal structure with the corresponding modified residue numbering.

The PTRAJ and CPPTRAJ software packages [24] were used to calculate root mean square deviation (RMSD) and root mean square deviation (RMSF). VMD software was used for visualizing the trajectories [25]. Complex modifications, as well as visualizations were conducted using the UCSF Chimera and Maestro software packages. For analyzing and plotting of graphs, the Origin data analysis tool was been used [26].

### 2.5. Sequence Alignment of Nedd4 Family

To validate the covalent hits for specificity against the Nedd4-1 enzyme, sequence alignment between the family of Nedd4-1 enzymes were evaluated. This family comprises of nine prominent members [27], all of which were aligned in an effort to compare the allosteric binding site region. This was carried out by downloading the FASTA sequences of the nine family members using the National Centre for Biotechnology information (NCBI) database [28]. These sequences included Nedd4-1 (NP46936), Nedd4-2 (Q96PU5), ITCHY (Q96J02), SMURF1 (Q9HCE7), SMURF2 (Q9HAU4), NEDL1 (Q76N89), NEDL2 (Q9P2P5), WWP1 (Q9H0M0), and WWP2 (O00308). Multiple sequence alignment was then completed using the UCSF software package [20].

## 3. Results and Discussion

### 3.1. Hit Compounds Analysis

A linear virtual screening strategy was used to search the ZINC database using both pharmacophore-based and docking-based screening. After pharmacophore-based screening, 3304 compounds from Zinc database were filtered by non-covalent docking. 114 compounds were then chosen based on their binding affinity to the Nedd4-1 and examined to identify whether the α, β-unsaturated ester group could be attached. Only the four compounds that met this requirement were retained for MD simulations and ADME investigations (Table 2). 

### 3.2. Screened Compounds Facilitate the Restriction of Nedd4-1 Structural Flexibility 

**Figure 4:**
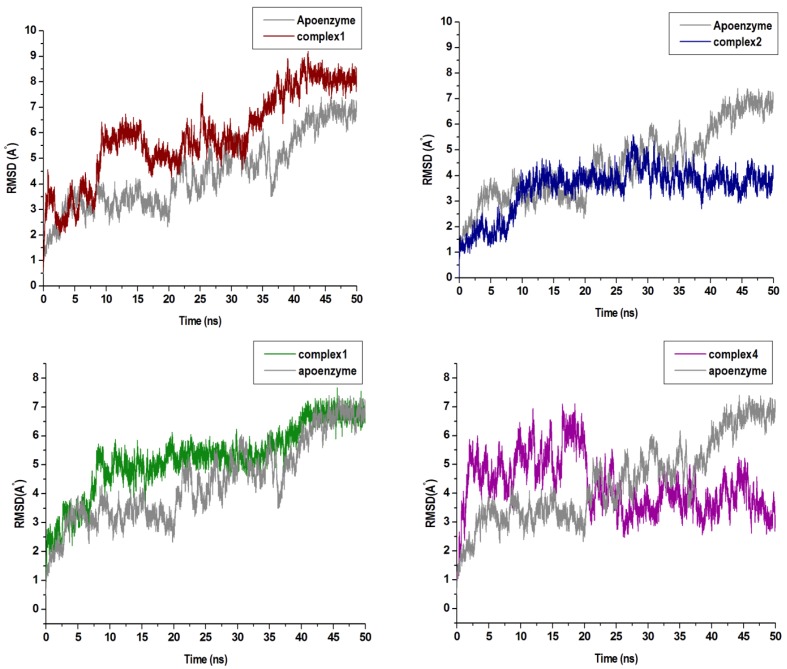
C-a backbone RMSD (Root-Mean-Square Deviation) plots of the four compounds in complex with Nedd4-1 when compared with the Apo enzyme.

The C-α RMSD and C-α RMSF values of backbone atoms with respect to their starting structures can appropriately evaluate the stability of a 3D protein structure. This can assist in the understanding of the protein dynamics and the equilibrium of the structures. In this respect, the variations in the residue positions were explored by calculating RMSD and RMSF across the duration of the MD simulation. The RMSD plots seen in Figure 4 show convergence of the overall structures at approximately 40 ns. Complex 1, 2, 3, and 4 refer to the enzyme bound to the respective compounds. The RMSD values of the complexes 1 and 3 were slightly higher than the free enzyme. In complex 4, the RMSD value was greater than the free enzyme at the start of the simulation, thereafter decreasing at 20 ns of the MD simulation period. However, in complex 2, the values were lower than the free enzyme throughout the simulation. This may have been a result of the stabilizing characteristics of compounds at the allosteric site of the compound. 

**Figure 5:**
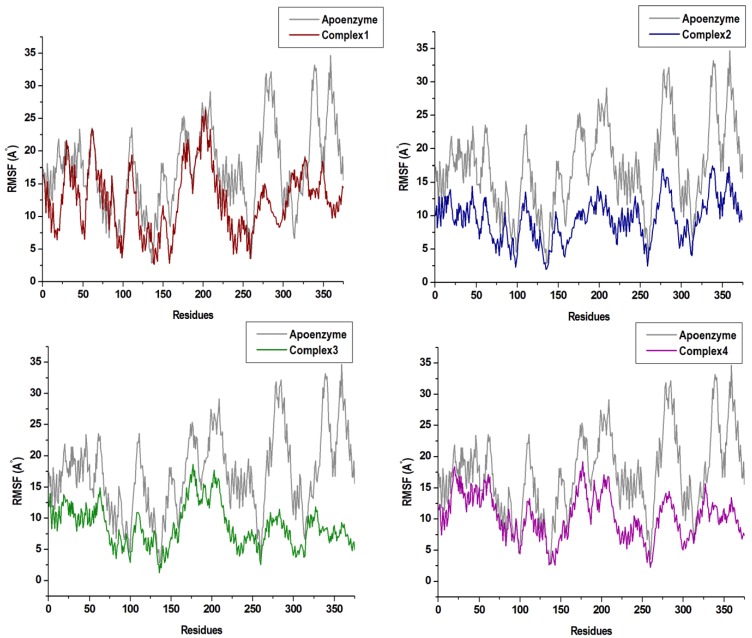
The RMSF plots of the four compounds in complex with Nedd4-1 in comparison with the Apo enzyme.

This was further supported by the RMSF analysis as seen in Figure 5, demonstrating that, although the overall fluctuations in the free enzyme were higher than those in all complexes, complexes 2 and 4 showed significant decline of RMSF. Specifically, in the catalytic region (amino acids 300–350), which confirms statements from previous studies elucidating the presence of the covalent ligand in the allosteric site of Nedd4-1 can affect its catalytic site, resulting in the full inhibition of the enzyme. To this effect, binding free energy investigations were carried out to achieve energy profiles of the studied complexes.

### 3.3. Comparative Binding Free Energy Profiles of Hit Compounds

The relative binding free energy (ΔG_bind_) of compound **1**, **2**, **3**, and **4** were computed using the Molecular Mechanics Generalized Born and Surface Area continuum solvation (MM/GBSA) approach [29]. The MMGBSA method is a popular approach that is often used to estimate the binding free energy of a small ligand to a biological macromolecule. MM/GBSA rely on molecular simulations of the ligand-protein complex to compute rigorous statistical-mechanical binding free energy within a specified force field [30]. The MM/GBSA approach has been successfully implemented to reproduce and rationalize experimental findings as well as to expand on results obtained from virtual screening and docking calculations [31]. To ascertain the binding affinity of the compounds and to establish the basis of potential inhibition against Nedd4-1, we calculated the binding free energy and per residue decomposition of each compound at the allosteric site of the enzyme, as seen in Table 3.

**Figure 6:**
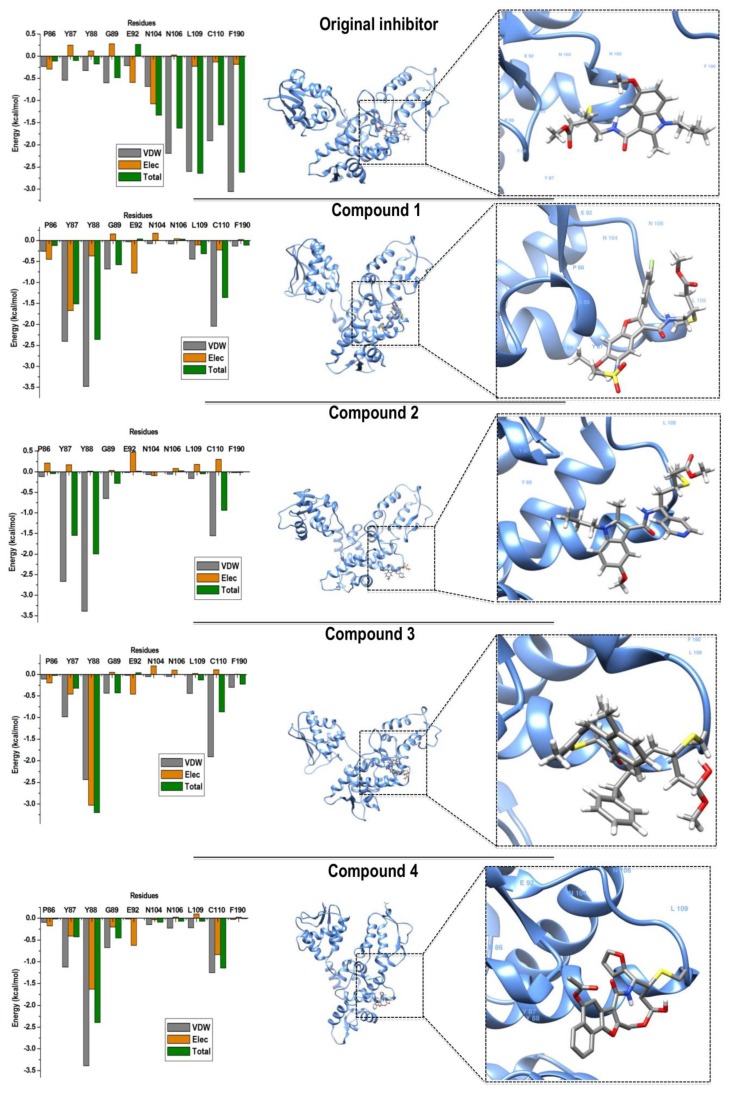
Per-residue decomposition analyses revealing individual energy contributions of allosteric site residues to the binding and stability of original compound and compounds **1**–**4**. Residues contributing the most to binding of all four compounds included TYR 87, TYR 88, and CYS 110.

Binding landscape analysis revealed that all four compounds bound in a similar conformation, with binding-site residues remaining common amongst all complexes, as seen in Figure 6.

Based on these results, the binding energies of compound **3** (−29.27 kcal/mol) and 4 (−28.20 kcal/mol) showed optimal binding when compared with compound **1** and **2** (−22.21 and −26.84 kcal/mol, respectively). The binding energy seen in complex 3 was attributed to elevated van der Waals and electrostatic energy between TYR 88 (TYR 605) and the methyl function group of compound **3**. A similar trend was observed between TYR 88 and the terminal benzene ring of compound **4**. Residues ASN 104 (ASN 621) and ASN 106 (ASN 623) contributed minimal interactions in all four complexes; this may have been due to the residue location at the outer edge of the binding-site region. Notably, the most favourable interactions with CYS 110 (CYS 627) were seen in complex 4, in which both van der Waals and electrostatic energies showed optimal ranges. The estimated binding free energies, although not absolute values as experimental values, are still reliable based on the binding region and residue interactions [13].

### 3.4. Predictive Pharmacokinetic Analysis of Hit Compounds

Hits that successfully passed the allocated filters, were then subjected to further ADME profile investigations using the SWISSADME online tool, as seen in Table 4 [32]. Our predictions revealed that compounds **1** and **3** possessed favorable pharmacokinetic profile but violated druglikeness. Although compound **2** showed high GIT absorption, it also violated the range of druglikeness. None of the identified compounds were shown to pass through the blood–brain barrier. This downfall may be alleviated by the encapsulation of the compounds within a drug delivery system to allow for passage [33]. Of the analyzed compounds, compound **4** fell into the accepted range of the six descriptor values of Lipinski’s Rule; it also exhibited better pharmacokinetic profile when compared with the other compounds. These results are in accordance with the molecular dynamic analyses, suggesting compound **4** as a strong hit for the inhibition of Nedd4-1.

### 3.5. Specifity Lead Compounds to the Nedd4-1 Enzyme

**Figure 7:**
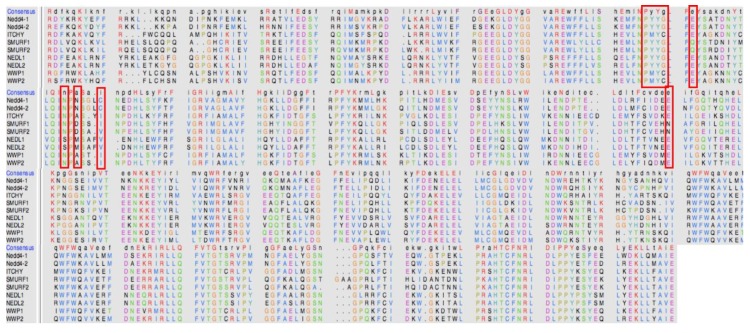
Sequence alignment of the nine members of the Nedd4 family.

To evaluate the specificity of compound **4** to the allosteric binding site of Nedd4-1, multiple sequence alignment was carried with the nine most prominent members of the Nedd4 family, as seen in Figure 7 [27]. Upon analysis of the alignment, it was evident that Nedd4-2 remained the most conserved with the Nedd4-1 amino acid sequence. The vital cysteine residue of the allosteric site remained conserved with only the Nedd4-2 sequence; however, ITCHY, SMURF1, WWP1, and WWP2 contained an isoleucine at residue 110, and SMURF2, WWP1 and WWP2 comprised of valine residues. This meant that the specify of the α, β-unsaturated ester group of compound **4** would have high affinities to not only Nedd4-1 but also to the Nedd4-2 enzyme. Previous studies also support our analysis; Nedd4-1 and Nedd4-2 are most phylogenetically-related and the overexpression of either enzyme may lead to dysfunctional metabolic processes [34,35]. It can be deduced that compound **4** may be specific to the allosteric site of the Nedd4-1 and Nedd4-2 enzymes.

## 4. Conclusions

Based on the detrimental role of the overexpression of Nedd4-1 in a multitude of diseases, it has proven to be a popular drug target. One characteristic of the enzyme is that it is covalently targeted. The use of computers in drug discovery aids in delivering new drug candidates at a faster rate whilst integrating cost efficiency. In this study, we paid special attention to the covalent inhibitory properties of the enzyme and identified, with the use of computational tools, potential allosteric inhibitors against this peculiar enzyme. Due to the challenges related to this task and the scarcity of the electrophilic moieties in the chemo-libraries, the non-covalent part of an existing covalent inhibitor of Nedd4-1 was used to generate a pharmacophore model and screen for novel inhibitor scaffolds of this enzyme. Of the screened compounds, four hits matched the criteria required for a covalent inhibitor. Molecular dynamic simulations and pharmacokinetic analysis were then utilized as computational validation of the compounds. Of the assessed data, compound **4** proved to be most appropriate as a covalent Nedd4-1 allosteric inhibitor, demonstrating both favourable binding to the allosteric site of the enzyme and promising pharmacokinetic profiling. The techniques implemented in this study were uniquely selected for their robustness and efficacy; they also enabled efficient translation to the medicinal chemistry domain. The method used in this study can be further implemented on a wide range of covalently inhibited enzymes, allowing for increased inhibitor potency and efficiency. The lead compound filtered from pharmacophore-based screening can be further biologically assessed for its efficiency and physiologic toxicity.

## Figures and Tables

**Table 1:** Original numbering of allosteric residues of the Nedd4-1 obtained from the original crystal structure with the corresponding residue numbering after running LEaP.

Allosteric Site Residues	
Original Residue Numbering	Modified Residue Numbering
PRO 603	PRO 86
TYR 604	TYR 87
TYR 605	TYR 88
GLY 606	GLY 89
GLU 609	GLU 92
ASN 621	ASN 104
ASN 623	ASN 106
LEU 626	LEU 109
CYS 627	CYS 110
PHE 707	PHE 190

**Table 2:** Four compounds that successfully passed the assigned filters. The connecting points with the covalent part of the model are highlighted in red.

Compound Number	Zinc ID or IUPAC ID	Chemical Structure	Docking Score (kcal/mol)
Original inhibitor	Methyl (2*E*)-4-[(1-cyclopentyl-5-methoxy-2-methylindol-3-yl) formamido] but-2-enoate	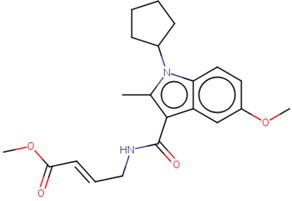	−7.6
**1**	Zinc83318266	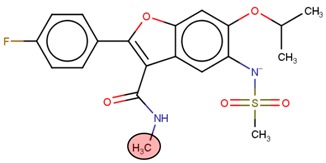	−8
**2**	Zinc78233917	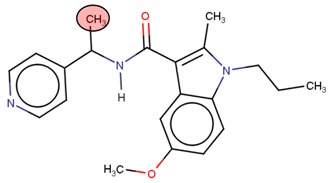	−7.3
**3**	Zinc09841012	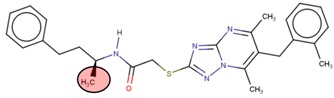	−7.7
**4**	Zinc00937975	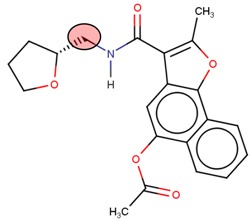	−7.4

**Table 3:** MMGBSA-based binding free energy profiles of original compound and compounds **1**–**4** at the allosteric site of Nedd4-1.

Energy Components (kcal/mol)					
Nedd4-1 Complex	ΔE_vdW_	ΔE_elec_	ΔG_gas_	ΔG_solv_	ΔG_bind_
Original inhibitor	−40.90 ± 4.20	−11.08 ± 3.06	−51.99 ± 5.77	19.39 ± 2.96	−**32.59** ± 3.98
Complex **1**	−34.58 ± 6.64	−10.25 ± 4.67	−44.83 ± 8.53	22.62 ± 4.86	−**22.21** ± 5.26
Complex **2**	−36.49 ± 3.08	−2.52 ± 3.18	−39.01 ± 4.31	12.17 ± 2.75	−**26.84** ± 2.92
Complex **3**	−34.18 ± 3.51	−21.23 ± 5.63	−55.41 ± 6.70	26.14 ± 5.23	−**29.27** ± 3.69
Complex **4**	−38.99 ± 4.12	−7.80 ± 5.90	−46.79 ± 6.98	18.59 ± 4.69	−**28.20** ± 4.19

Bold: highlight the total binding free energies and differentiate them from the components of the total values.

**Table 4:** ADME profiles of the top ranked hits. GIT and BBB stand for gastrointestinal absorption and blood–brain barrier permeability, respectively.

Compounds	Molecular Weight (g/mol)	Lipophilicity (iLOGP)	GIT Absorption	BBB Permeability	Synthetic Accessibility	Bioavailability Score	Druglikeness (Lipinski)
Original Inhibitor	370.44	3.71	High	Yes	2.92	0.55	Yes
**1**	506.54	3.45	Low	No	4.51	0.55	No
**2**	437.53	3.79	High	No	3.56	0.55	No
**3**	562.75	1.78	Low	No	5.19	0.55	No
**4**	451.47	3.88	High	No	4.38	0.55	Yes
